# Genetic diversity and allele frequencies of *Plasmodium falciparum msp1* and *msp2* in parasite isolates from Bioko Island, Equatorial Guinea

**DOI:** 10.1186/s12936-018-2611-z

**Published:** 2018-12-07

**Authors:** Jiang-Tao Chen, Jian Li, Guang-Cai Zha, Guang Huang, Zhi-Xiu Huang, Dong-De Xie, Xia Zhou, Huan-Tong Mo, Juan Urbano Monsuy Eyi, Rocio Apicante Matesa, Maximo Miko Ondo Obono, Shan Li, Xiang-Zhi Liu, Min Lin

**Affiliations:** 1 Laboratory Medical Center, Huizhou Municipal Central Hospital, Huizhou, Guangdong Province People’s Republic of China; 2 The Chinese Medical Aid Team to the Republic of Equatorial Guinea, Guangzhou, Guangdong Province People’s Republic of China; 3 Institute of Basic Medical Sciences, Hubei University of Medicine, Shiyan, Hubei People’s Republic of China; 4 School of Food Engineering and Biotechnology, Hanshan Normal University, Chaozhou, Guangdong Province People’s Republic of China; 5 Laboratory Medical Center, Chaozhou People’s Hospital, Shantou University Medical College, Chaozhou, Guangdong Province People’s Republic of China; 6 Department of Medical Laboratory, Malabo Regional Hospital, Malabo, Equatorial Guinea

**Keywords:** *Plasmodium falciparum*, Bioko Island, Genetic diversity, Multiplicity of infection (MOI)

## Abstract

**Background:**

Malaria is still a serious public health problem on Bioko Island (Equatorial Guinea), although the number of annual cases has been greatly reduced since 2004 through the Bioko Island Malaria Control Project (BIMCP). A better understanding of malaria parasite population diversity and transmission dynamics is critical for assessing the effectiveness of malaria control measures. The objective of this study is to investigate the genetic diversity of *Plasmodium falciparum* populations and multiplicity of infection (MOI) on Bioko Island 7 years after BIMCP.

**Methods:**

A total of 181 patients with uncomplicated *P. falciparum* malaria diagnosed with microscopy were collected from Bioko Island from January 2011 to December 2014. Parasite DNA was extracted using chelex-100 and species were identified using a real-time PCR followed by high-resolution melting. *Plasmodium falciparum msp1* and *msp2* allelic families were determined using nested PCR.

**Results:**

Three *msp1* alleles (K1, MAD20, and RO33) and two *msp*2 alleles (FC27 and 3D7) were analysed in all samples. In *msp1*, the MAD20 allelic family was predominant with 96.69% (175/178) followed respectively by the K1 allelic family with 96.07% (171/178) and R033 allelic family with 70.78% (126/178). In *msp2*, the FC27 allelic family was the most frequently detected with 97.69% (169/173) compared to 3D7 with 72.25% (125/173). Twenty-six different alleles were observed in *msp1* with 9 alleles for K1, 9 alleles for MAD20 and 8 alleles for R033. In *msp2*, 25 individual alleles were detected with 5 alleles for FC27 and 20 alleles for 3D7. The overall MOI was 5.51 with respectively 3.5 and 2.01 for *msp1* and *msp2*. A significant increase in overall MOI was correlated with the age group of the patients (*P *= 0.026) or parasite densities (*P *= 0.04).

**Conclusions:**

The present data showed high genetic diversity and MOI values among the *P. falciparum* population in the study, reflecting both the high endemic level and malaria transmission on Bioko Island. These data provide valuable information for surveillance of *P. falciparum* infection and for assessing the appropriateness of the current malarial control strategies in the endemic area.

## Background

Despite substantial efforts to eliminate or control malaria, this disease remains the leading cause of morbidity and mortality worldwide [1]. Bioko Island, Equatorial Guinea, has historically high malaria transmission [2]. For effective control of malaria, many measures have been deployed since 2004 through the Bioko Island Malaria Control Project (BIMCP), including island-wide indoor residual spraying (IRS), long-lasting insecticide-treated nets (LLIN) and artemisinin-based combination therapy (ACT) [2]. Although these control measures have resulted in a substantial decrease in malaria infection on Bioko Island, the disease is still endemic, with populations in some areas remaining at high risk of infection [2, 3].

Genotyping of malaria parasite populations remains an important tool to determine the types and number of parasite clones in an infection. In molecular epidemiological studies of malaria, this approach is used to investigate the genetic diversity of infections with consideration of various factors, including transmission intensity and host immunity [1]. The most widely used techniques for genotyping malaria infections are based on amplification by PCR of the polymorphic genes encoding the merozoite surface proteins 1 (MSP-1) and 2 (MSP-2) [2, 4, 5]. MSP1 and MSP2 are two major surface proteins of merozoites during the erythrocytic stage [6, 7]. MSP1 is a 190 kDa surface protein encoded by the *msp1* gene located on chromosome 9 and contains 17 blocks of sequences flanked by conserved regions [5, 6]. This protein is a major target of immune responses and is considered a noteworthy candidate for the development of erythrocytic phase malaria vaccines [8, 9]. Block 2, which is the most polymorphic part of MSP1, is grouped into three allelic families namely K1, MDA20 and R033 [10]. MSP2 is a glycoprotein encoded by the *msp2* gene located on chromosome 2 and is composed of five blocks, of which the central block 3 is the most polymorphic. The alleles of *msp2* are commonly divided into FC27 and 3D7 [11].

Several studies have linked high multiplicity of infection (MOI) to the severity of malaria, especially in areas with high transmission rates [12]. In this regard, determination of MOI in a highly endemic area such as Bioko Island is crucial because it predicts a clinical course target population that requires increased attention (e.g., certain age groups). Regular molecular epidemiological surveys that monitor the genetic diversity of *P. falciparum* populations in the region as well as worldwide and link parasite genotypes to the disease phenotypes are crucially important [1, 13, 14]. There is very limited information on *msp*1 and *msp*2 genetic diversity on Bioko Island, Equatorial Guinea. This study aimed to characterize the genetic diversity of *P. falciparum* populations and MOI in malaria parasites isolated from symptomatic patients on Bioko Island 7 years after BIMCP.

## Methods

### Study site

**Fig. 1:**
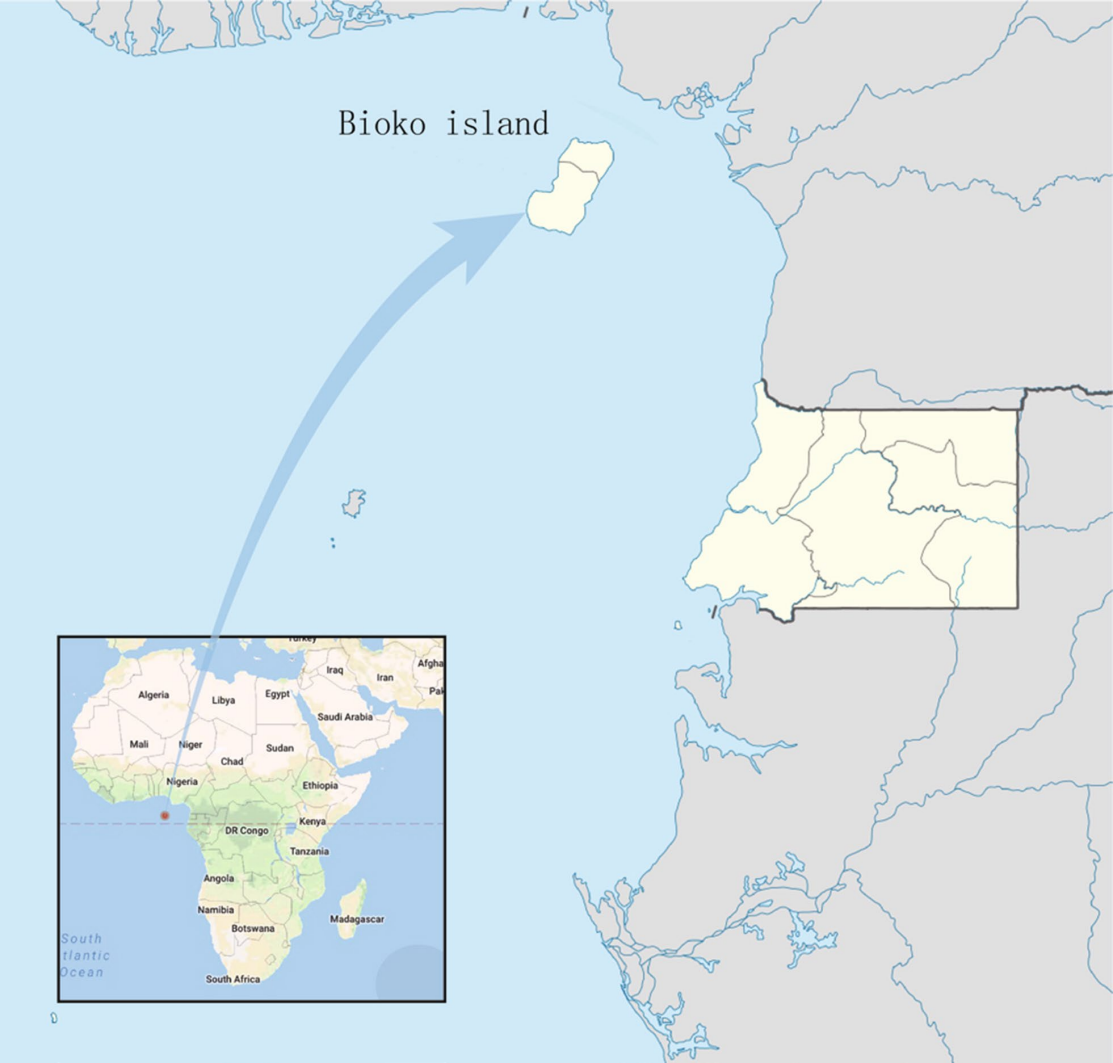
Map of Bioko Island of Equatorial Guinea

The study was carried out in the clinic of the Chinese medical aid team to the Republic of Equatorial Guinea, Malabo Regional Hospital in Malabo, located on the north coast of Bioko Island, Equatorial Guinea. This study was approved by the ethics committees of Malabo Regional Hospital. Bioko Island, the largest island of Equatorial Guinea, is located in the Gulf of Guinea, approximately 100 km off the coast of southern Nigeria and 160 km northwest of continental Equatorial Guinea (Fig. 1). The island has a population of 334,463 inhabitants (2015 census, of which approximately 90% live in Malabo, the capital city) and a humid tropical environment. The launch of the BIMCP has had a marked impact on malaria transmission, and malaria due to *P. falciparum* is still the major public health problem on the island [2]. The entomological inoculation rates (EIRs) in Bioko Island ranged from 163 to 840, with the outdoor EIRs reaching more than 900 infective mosquito bites yearly and a malaria prevalence of 52% under the age of 5 years [2].

### Study population and blood sample collection

A total of 181 blood spot samples were collected from the patients with uncomplicated malaria enrolled during therapeutic efficacy monitoring of artesunate + amodiaquine (Guilin Pharmaceutical Co., Ltd., Shanghai, China) between January 2011 and December 2014. Included patients were aged between 4 months and 80 years, and were residents on Bioko Island. Malaria patients were classified into uncomplicated malaria states according to the WHO criteria [15], which were defined as positive smear for *P. falciparum* and presence of fever (≥ 37.5 °C).

Consent was obtained from all participating subjects or their parents. Dried blood spots were collected on day zero of enrollment through finger prick bleeding spotted onto Whatman 903^®^ filter paper (GE Healthcare, Pittsburgh, USA) for future use. Microscopy thick and thin blood film slides were prepared using 10% Giemsa solution for 30 min. The stained slides were examined under a light microscope using 100 × oil immersions by an experienced laboratory technician. Parasite density was calculated per 200 white blood cells (WBC) assuming 8000 WBC/μl of blood stage. Based on the microscopy results, the study subjects were classified into three groups: group with low parasite density (< 1000 asexual parasites/μl), group with moderate parasite density (1000–9999 asexual parasites/μl) and group with high parasite density (≥ 10,000 asexual parasites/μl). For quality control, archived malaria-positive microslides were re-examined, and parasite density was recorded; *Plasmodium species* were identified by a real-time PCR followed by high-resolution melting (HRM) [16]. The pGEM-T standard plasmids of four human *Plasmodium* species including *P. falciparum*, *Plasmodium ovale*, *Plasmodium malariae* and *Plasmodium vivax*, which were kindly provided by Dr. Cao J (Jiangsu Institute of Parasitic Diseases, Wuxi, Jiangsu Province, China) [16], were used as control.

### Extraction of parasite DNA

Genomic DNA was extracted from the dried blood spots using 0.5% saponin (Sigma-Aldrich, Taufkirchen, Germany) to free parasites from red blood cells followed by the Chelex^®^ 100 (Bio-Rad Laboratories, CA, USA) method as previously described [1, 16] and stored at − 20 °C.

### Allelic genotyping of the *Plasmodium falciparum msp1* and *msp2* genes

Primers specific for the polymorphic regions of *msp1* (block 2) and *msp2* (block 3) were previously described [12, 17]. The polymorphic allelic families of the *msp1* (K1, MAD20, and RO33) and *msp2* (FC27 and 3D7) genes were amplified with a nested PCR amplification (Bio-Rad Mini MJ thermal cycler). For the first reaction, 1.0 μl of DNA was amplified with 12.5 μl 2× Taq PCR MasterMix (Aidlab Biotechnologies Co., Ltd., Beijing, China), 1 μl forward primer (10 μM), 1 μl reverse primer (10 μM), and sterile ultrapure water to a final volume of 25 μl. For the second round of PCR, 1.0 μl of first-round PCR products was amplified with a 50 μl reaction system, which included 25 μl 2× Taq PCR MasterMix (Aidlab Biotechnologies Co., Ltd., Beijing, China), 2.0 μl forward primer (10 μM), 2.0 μl reverse primer (10 μM), and water (up to 50 μl). The primary PCR amplification of the *msp1* and *msp2* genes comprised an initial step of 94 °C for 3 min followed by 30 cycles of 94 °C for 30 s, 55 °C for 30 s, 72 °C for 1 min, and a final extension of 72 °C for 5 min. The nested PCR cycling parameters of the second round were the same as those of the primary reaction. Allelic specific positive control and DNA negative control were included in each set of reactions.

### Detection of alleles

The resultant PCR products were stained with SYBR^®^ Green I nucleic acid gel stain (Dongsheng Biotech, Guangzhou, China) and resolved by gel electrophoresis in 2.5% agarose gel. DNA sizes were determined using a low molecular weight ladder marker (25–700 bp, Dongsheng Biotech, Guangzhou, China) and photographed using a Tanon 2500/2500R Gel Imaging System (Tanon Science & Technology Co., Ltd., Shanghai, China). Alleles of *msp1* and *msp2* were categorized according to their molecular weights and regarded as different when the molecular weight difference was equal to or greater than 20 bp.

### Multiplicity of infection

Multiplicity of infection (MOI) was determined by calculating the number of different alleles at each locus; single infections were those with only one allele per locus at all of the genotyped loci. Multiclonal infections were defined as those having more than one allele in at least one locus out of the loci genotyped [18, 19].

### Statistical analysis

All statistical analyses were performed using the software Statistical Package for Social Sciences version 17.0 (SPSS, Inc., Chicago, IL, USA). The *msp1* and *msp2* allelic frequency was calculated. The mean MOI was calculated for *msp1*, *msp2* and overall MOI. The overall MOI was determined by dividing the total number of alleles detected in both *msp1* and *msp2* by the total number of samples. The proportions of allele comparisons were assessed by Chi square tests, and normally distributed continuous data were assessed by analysis of variance (ANOVA). The Spearman’s rank correlation coefficient was calculated to evaluate relationships between MOI, parasite densities or age groups in the patients. Statistical significance was defined as *P* < 0.05.

## Results

### General characteristics

Overall, 181 patients with uncomplicated malaria diagnosed with microscopy were enrolled in the study. They were confirmed with *P. falciparum* monoinfection by PCR-HRM [16]. Among these patients, 53.59% (97/181) were males and 46.41% (84/181) were females. Six (3.31%, 6/181) were children younger than 5 years of age; 47 (25.97%, 47/181) were children from 5 to 19 years of age; 87 (48.07%, 87/181) were patients between 20 and 39 years old; and 41 (22.65%, 41/181) patients were 40 years old or older. The mean body axillary temperature measured prior to blood sampling was 38.7 °C (± 1.3) and the mean parasite count was 64,804 parasites (420–816,300) per μl of blood.

### Frequency of *msp1* and *msp2* allelic families

Among these 181 *P. falciparum* isolates, 178 (98.34%) samples showed successful amplification of *msp1*, and 173 (95.58%) showed amplification of *msp2*. Detailed information on *msp1* and *msp2* is shown in Table 1. For *msp1*, the MAD20 allelic family was predominant at 98.31% (175/178), followed by the K1 allelic family at 96.07% (171/178) and the R033 allelic family at 70.78% (126/178). Almost all samples positive for *msp1* (98.88%, 176/178) were classified as polyclonal infections with K1/MAD20, K1/R033, MAD20/R033 and K1/MAD20/R033, which represented 27.6, 1.7, 2.8 and 65.2%, respectively. MAD20 was the only allelic type with a monoclonal infection frequency of 1.10% (2/181). For *msp2*, the FC27 allelic family was the most frequently detected, with 97.68% (169/173), compared to 3D7 with 72.25% (125/173).

**Table 1:** Genetic diversity of *Plasmodium falciparum msp1* and *msp2* among age groups of symptomatic patients on Bioko Island, Equatorial Guinea (2011–2014)

Gene	Allelic type	Age group (years)				Total
		< 5	5–19	20–39	≥ 40	
		*n* (%)	*n* (%)	*n* (%)	*n* (%)	*n* (%)
		(*n *= 6)	(*n *= 47)	(*n *= 88)	(*n *= 40)	(*n *= 181)
*msp1*	K1	0 (0)	0 (0)	0 (0)	0 (0)	0 (0)
	MAD20	0 (0)	0 (0)	0 (0)	2 (5.0)	2 (1.1)
	RO33	0 (0)	0 (0)	0 (0)	0 (0)	0 (0)
	K1/MAD20	0 (0)	5 (10.6)	28 (31.8)	17 (42.5)	50 (27.6)
	K1/RO33	1 (26.7)	0 (0)	1 (1.1)	1 (2.5)	3 (1.7)
	MAD20/RO33	0 (0)	1 (0.55)	1 (1.1)	3 (7.5)	5 (2.8)
	K1/MAD20/RO33	5 (83.3)	41 (87.2)	55 (62.5)	17 (42.5)	118 (65.2)
	Total	6 (100)	47 (100)	85 (96.6)	38 (95)	178 (97.2)
	MOI	3.33	3.81	3.59	3.03	3.5
*msp2*	3D7	1 (26.7)	7 (14.9)	26 (29.5)	14 (35)	48 (26.5)
	FC27	1 (26.7)	1 (2.1)	1 (1.1)	1 (2.5)	4 (2.2)
	3D7/FC27	4 (66.7)	37 (78.7)	55 (62.5)	25 (62.5)	121 (66.9)
	Total	6 (100)	45 (95.7)	82 (93.2)	40 (100)	173 (95.6)
	MOI	1.83	2.31	2.01	2.1	2.01
Multiclonal isolates		6 (100)	47 (100)	87 (98.9)	41 (100)	181 (100)
Overall MOI		5.17	6.02	5.53	5.13	5.51

*n* number of individuals, *MOI* multiplicity of infection

### Genetic diversity and allelic frequency

**Fig. 2:**
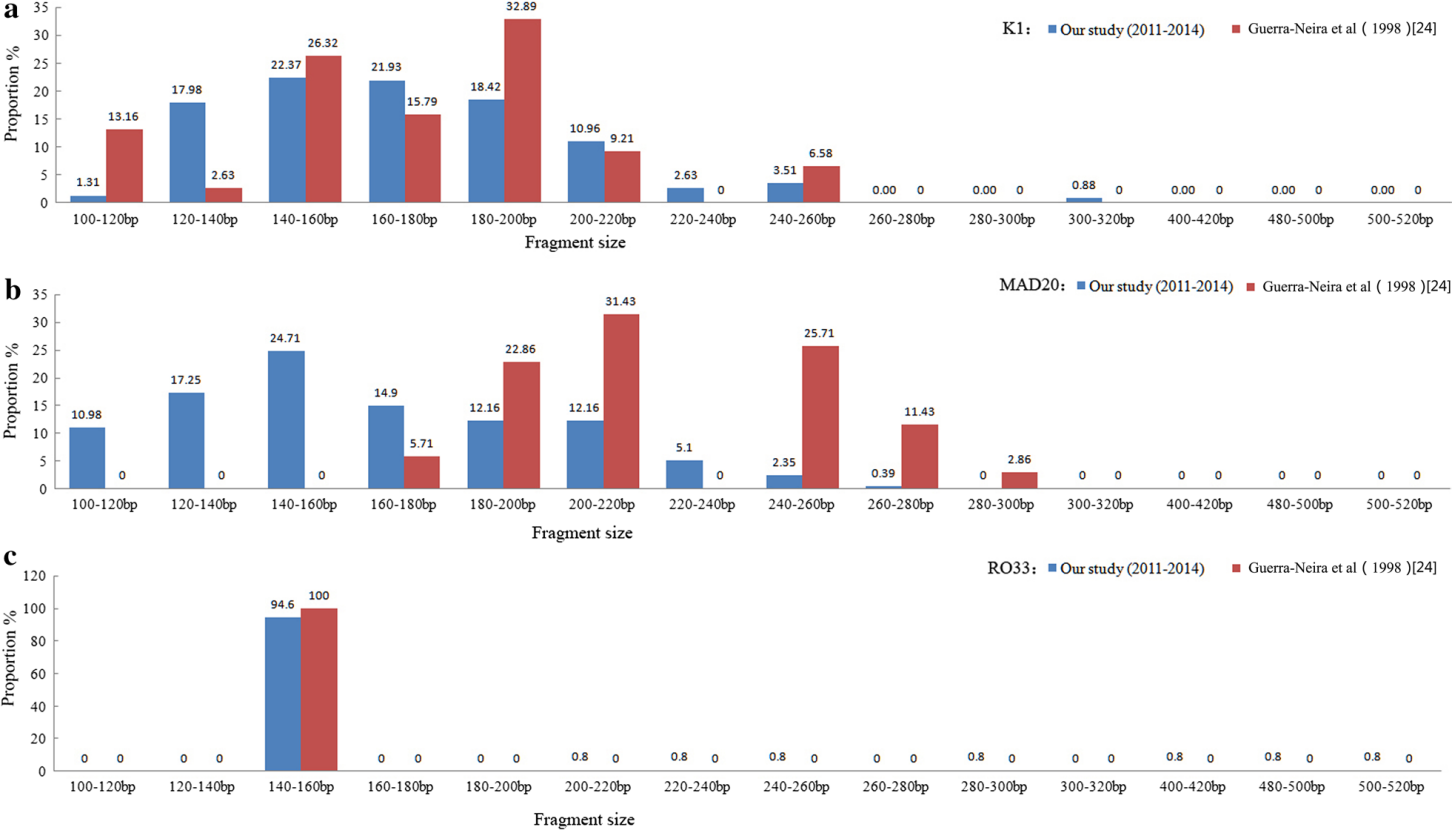
Genetic diversity of *msp1* of *Plasmodium falciparum* isolates from Equatorial Guinea (**a**) K1 allele distribution (**b**) MAD20 allele distribution (**c**) RO33 allele distribution

**Fig. 3:**
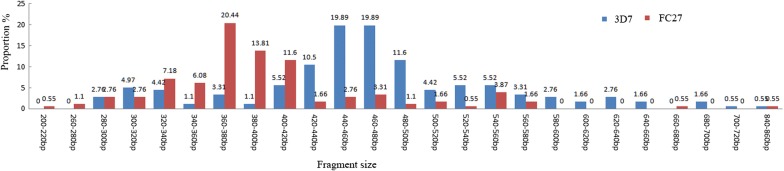
Genetic diversity of *msp2* of *Plasmodium falciparum* isolates from Equatorial Guinea

Twenty-six different alleles were observed in *msp1* including 9 K1 type alleles with a size ranging from 100 to 320 bp, 9 MAD20 type alleles (100–280 bp) and 8 R033 type alleles (140–520 bp). For the 140–160 bp fragment range, the frequencies of RO33 were significantly higher than those of K1 (*P *< 0.001) and MAD20 (*P *< 0.001). The different fragment sizes of each family of *msp1* were shown in Fig. 2a–c. Based on *msp2* gene analysis, a total of 25 individual alleles including 20 3D7 type alleles with a size ranging from 200 to 860 bp and 5 FC27 type alleles (320–420 bp) were identified (Fig. 3). One obvious difference was that the high-frequency allele size of FC27 was mainly 320–420 bp with 5 allele sizes (320–340 bp; 340–360 bp; 360–380 bp; 380–400 bp; 400–420 bp), where as the high-frequency allele size of 3D7 was mostly 400–560 bp with 7 allele sizes (400–420 bp; 420–440 bp; 440–460 bp; 460–480 bp; 500–520 bp; 520–540 bp; 540–560 bp). The allele distribution for both the FC27 and 3D7 families is illustrated in Fig. 3.

### MOI, parasite density and different distribution of allelic families across different age groups

Almost all malaria patients in all age groups were found to have multiclonal infections. The MOI values for *msp1* and *msp2* are summarized in Table 1. There was a significant positive correlation between the overall MOI number and age groups (Spearman rank coefficient = − 0.209; *P *= 0.005). When the *msp1* and *msp2* genes were considered separately, the MOI values were 3.5 and 2.01, respectively. There was a significant correlation between MOI for *msp1* and age groups of patients (Spearman rank coefficient = − 0.286; *P *< 0.001). However, no significant correlation between MOI for *msp2* and age groups (Spearman rank coefficient = − 0.05; *P *= 0.515) was found. As shown in Table 2, the distribution of different *msp1* and *msp2* allelic families and their different combinations showed a positive impact on the parasite density. The results show that the overall MOI were significantly different among groups with different parasite densities (F = 5.761, *P *= 0.004). The overall MOI in the group with low parasite density (< 1000 parasites/μl) was significantly lower than that of the groups with moderate (*P *= 0.008) or high parasite density (*P *= 0.001).

**Table 2:** Genetic diversity of *Plasmodium falciparum msp1* and *msp2* among parasitaemic groups of symptomatic patients on Bioko Island, Equatorial Guinea (2011–2014)

Gene	Allelic type	Parasite density (no. of parasites/μl)		
		< 1000	1000–9999	≥ 10,000 (%)
		*n* (%)	*n* (%)	*n* (%)
		(*n *= 14)	(*n *= 74)	(*n *= 93)
*msp1*	MAD20	1 (7.1)	0	1 (1.1)
	K1/MAD20	3 (21.4)	22 (29.7)	25 (27.8)
	K1/RO33	1 (7.1)	1 (1.4)	1 (1.1)
	MAD20/RO33	0	4 (5.4)	1 (1.1)
	K1/MAD20/RO33	9 (64.3)	46 (62.2)	63 (70)
	Total	13 (92.9)	73 (98.6)	90 (49.72)
	MOI	3.07	3.63	3.48
*msp2*	3D7	3 (21.4)	25 (33.8)	20 (21.5)
	FC27	1 (7.1)	2 (2.7)	1 (1.1)
	3D7/FC27	7 (50)	45 (60.8)	69 (74.2)
	Total	7 (50)	45 (60.8)	69 (74.2)
	MOI	1.73	1.90	2.31
Multiclonal isolates		14 (100)	74 (100)	93 (100)
Overall MOI		4.43	5.51	5.77

*n* number of individuals, *MOI* multiplicity of infection

## Discussion

The genetic diversity of *P. falciparum* parasites impacts malaria transmission and malaria control strategies. Therefore, it is important to resolve the genetic population structure of *P. falciparum* parasites in epidemic areas. However, very few studies have investigated the genetic diversity of *msp1* and *msp2* in malaria parasites circulating in many endemic countries, including Equatorial Guinea. This study aimed to evaluate the genetic diversity and allelic frequency of *msp1* and *msp2* in malaria parasites isolated from the patients with uncomplicated malaria on Bioko Island.

For the *msp1* and *msp2* genes, the high diversity is compatible with the high level of malaria transmission on the island. A total of 26 and 25 different alleles for *msp1* and *msp2*, respectively, were obtained from the parasite isolates. This genetic diversity was higher than that in the neighbouring countries of Central Africa, such as the south-western region of Nigeria (*msp1*: 5; *msp2*: 15) [20], Brazzaville of the Republic of Congo (*msp1*: 15; *msp2*: 20) [21] and Bangui of Central African Republic (CAR) (*msp1*: 17; *msp2*: 25) [22], but lower than Gabon (*msp1*: 39; *msp2*: 27) [23]. In order to better understanding the changing of parasite population diversity on Bioko Island after implementation of BIMCP, a comparison was made between the findings of this study with those of Guerra-Neira et al. [24] (a sample collection from Bioko in 1998) (Fig. 2a–c), which was the only record of *P. falciparum msp1* and *msp2* since 13 years ago. In their report, only 16 and 8 alleles, respectively, were identified [20]. MAD20 was the predominant allelic family in this study, contrary to their report, where the K1 allelic family was reported as the most common [24]. As shown in Fig. 2b, the allele distribution for MAD20 was significantly different from the allele distribution reported by Guerra-Neira et al. [24]. Although this comparison was somewhat flawed because Guerra-Neira et al. used different criteria for their study (i.e., isolates from asymptomatic individuals; individuals aged 10 years and below; three villages on Bioko Island), as compared to the criteria used here (i.e., symptomatic patients; aged 4 months to 80 years; one site only in Malabo), the findings still indicate a trend of an increasing allelic frequency of the MAD20 family on Bioko between 1998 and 2014. In addition, the higher MOI recorded in this study also depicts the complexity of the circulating parasite population. This changing genetic makeup of *P. falciparum* may be due to high transmission intensity and the possible impact of different control measures through BIMCP, mainly IRS, introduction of LLIN and changes in anti-malarial drugs [2, 25].

A high prevalence of multiclonal infection (98.34%) was observed in the investigation, with an overall MOI of 5.46. This value is higher than that reported in many African countries, including southern Ghana (1.17–1.48) [13], the Republic of Congo (1.7) [23], Nigeria (2.6–2.8) [21, 26], Gabon (4.0) [23], and southwest Ethiopia (1.8) [27]. The difference in MOI can be explained by the differences in geographical areas, intensity of malaria transmission and other factors, such as the difference in age of study population and mean parasite density in the study population. Notably, the current study was conducted after the implementation of ACT on Bioko Island [25].

A positive correlation between the overall MOI number and age of patients was found in the study. The findings indicate a multiplicity peak in 5- to 19-year-old patients, with overall MOI decreasing slowly afterwards. This finding was consistent with several previous reports [20, 28, 29]. Previous studies regarding the variation in MOI with age have suggested that the influence of age on the MOI is highly affected by endemicity of malaria, which is probably a reflection of the development of anti-parasite specific immunity [30]. Interestingly, when parasite density was categorized into three groups (< 1000, 1000–9999 and > 9999 parasites/μl), the genetic diversity was significantly influenced by parasite density (*P *< 0.05) (Table 2). These results are consistent with many reports demonstrating that high parasite densities increase the probability of detecting concurrent clones in an individual [31, 32]. This finding also indicates that high malaria transmission and parasite density may have a strong association with the genetic diversity of *P. falciparum* on Bioko Island.

## Conclusions

This study provides foundational information on the genetic diversity of *P. falciparum* (*msp1* and *msp2*) after the deployment of many malaria control measures through the BIMCP since 2004. Malaria caused by *P. falciparum* on the island was primarily multiple infections with generally high parasite variation, together with a high predominance of the K1 and MAD20 allelic families of *msp1* and 3D7 and FC27 families of *msp2*. These findings also indicate that a methodical exploration of malaria prevalence, with full-scale drug resistance surveillance, is essential for effective malaria prevention and eradication countermeasures.

## Abbreviations

BIMCP: Bioko Island Malaria Control Project
IRS: island-wide indoor residual spraying
LLIN: long-lasting insecticide-treated nets
ACT: artemisinin-based combination therapy
*msp1*: merozoite surface protein 1
*msp2*: merozoite surface protein 2
kDa: kilodalton
EIR: entomological inoculation rate
DNA: deoxyribonucleic acid
PCR: polymerase chain reaction
UV: ultraviolet
MOI: multiplicity of infection
